# Effects of β-Blockers on the Sympathetic and Cytokines Storms in Covid-19

**DOI:** 10.3389/fimmu.2021.749291

**Published:** 2021-11-11

**Authors:** Hayder M. Al-kuraishy, Ali Ismail Al-Gareeb, Gomaa Mostafa-Hedeab, Keneth Iceland Kasozi, Gerald Zirintunda, Akhmed Aslam, Mamdouh Allahyani, Susan Christina Welburn, Gaber El-Saber Batiha

**Affiliations:** ^1^ Department of Clinical Pharmacology and Medicine, College of Medicine, ALmustansiriyia University, Baghdad, Iraq; ^2^ Pharmacology Department, Health Sciences Research Unit, Medical College, Jouf University, Sakaka, Saudi Arabia; ^3^ Infection Medicine, Deanery of Biomedical Sciences, College of Medicine and Veterinary Medicine, The University of Edinburgh, Edinburgh, United Kingdom; ^4^ School of Medicine, Kabale Unviersity, Kabale, Uganda; ^5^ Department of Animal Production and Management, Faculty of Agriculture and Animal Sciences, Busitema University, Tororo, Uganda; ^6^ Laboratory Medicine, Faculty of Applied Medical Sciences, Umm Al-Qura University, Makkah, Saudi Arabia; ^7^ Department of Clinical Laboratory Sciences, College of Applied Medical Sciences, Taif University, Taif, Saudi Arabia; ^8^ Zhejiang University-University of Edinburgh Institute, Zhejiang University School of Medicine, Zhejiang University, Haining, China; ^9^ Department of Pharmacology and Therapeutics, Faculty of Veterinary Medicine, Damanhour University, Damanhour, Egypt

**Keywords:** SARS-CoV-2, cytokine storm, sympathetic storm, pharmacology and Covid, Beta blockers

## Abstract

Severe acute respiratory syndrome coronavirus 2 (SARS-CoV-2) is a causative virus in the development of coronavirus disease 2019 (Covid-19) pandemic. Respiratory manifestations of SARS-CoV-2 infection such as acute lung injury (ALI) and acute respiratory distress syndrome (ARDS) leads to hypoxia, oxidative stress, and sympatho-activation and in severe cases leads to sympathetic storm (SS). On the other hand, an exaggerated immune response to the SARS-CoV-2 invasion may lead to uncontrolled release of pro-inflammatory cytokine development of cytokine storm (CS). In Covid-19, there are interactive interactions between CS and SS in the development of multi-organ failure (MOF). Interestingly, cutting the bridge between CS and SS by anti-inflammatory and anti-adrenergic agents may mitigate complications that are induced by SARS-CoV-2 infection in severely affected Covid-19 patients. The potential mechanisms of SS in Covid-19 are through different pathways such as hypoxia, which activate the central sympathetic center through carotid bodies chemosensory input and induced pro-inflammatory cytokines, which cross the blood-brain barrier and activation of the sympathetic center. β2-receptors signaling pathway play a crucial role in the production of pro-inflammatory cytokines, macrophage activation, and B-cells for the production of antibodies with inflammation exacerbation. β-blockers have anti-inflammatory effects through reduction release of pro-inflammatory cytokines with inhibition of NF-κB. In conclusion, β-blockers interrupt this interaction through inhibition of several mediators of CS and SS with prevention development of neural-cytokine loop in SARS-CoV-2 infection. Evidence from this study triggers an idea for future prospective studies to confirm the potential role of β-blockers in the management of Covid-19.

## Introduction

It is well-known in recent times that severe acute respiratory syndrome coronavirus 2 (SARS-CoV-2) is a causative virus in the development of coronavirus disease 2019 (Covid-19) pandemic (1). This disease was initially documented in the Wuhan province of China (2). The SARS-COV-2 virus is highly infective with about 15% of the patients require hospitalization and 5% may need intensive care (3). Approximately half of the Covid-19 patients taken to intensive care units (ICU) die due to various complications associated with acute respiratory distress syndrome (ARDS) (4). The severe Covid-19 complications include respiratory failure, cardiac arrhythmias, acute kidney injury, and stroke (5). Respiratory failure is a result of acute lung injury (ALI) and ARDS (6). The respiratory system signs lead to hypoxia, oxidative stress, and sympatho-activation and in severe cases lead to sympathetic storm (SS) (7). SS is characterized by recurrent episodes of hyperhidrosis, hypertension, tachycardia, tachypnea, and hyperthermia (8).

On the other hand, exaggerated immune response to the SARS-CoV-2 invasion may lead to the production of various inflammatory substances (9). There may be an uncontrolled release of pro-inflammatory cytokines such as interleukins (IL-6, IL-1β, IL-8), tumor necrosis factor-alpha (TNF-α), and chemokines that together lead to the development of cytokine storm (CS) (10).

In Covid-19, there is interactive interaction between CS and SS in the development of multi-organ failure (MOF) and life-threatening complications (11). However, cutting the bridge between CS and SS by anti-inflammatory and anti-adrenergic agents may mitigate complications that are induced by SARS-CoV-2 infection in severely affected Covid-19 patients (12).

Anti-adrenergic β-blockers are a class of medications used in the management of cardiovascular disorders such as arrhythmia, acute coronary syndrome, and hypertension as well as other disorders like tremor and anxiety (13). β-blockers are either selective (block β1 or β2) or non-selective (block both β1 and β2). β-blockers reduce sympathetic stimulation-mediated by adrenalin and noradrenalin on β receptors (13). β1 receptors are located mainly on the heart and kidney while, β2 receptors are expressed primarily in the lungs, vascular smooth muscles, and gastrointestinal tract (14).

The objective of the present study was to summarize the current updates in the discussed topic and demonstrates the potential guidance for the usage of beta-blocker on the SS and CS in Covid-19.

## β-Blockers and Sympathetic Storm in Covid-19

It has been reported that β-blockers such as propranolol, metoprolol, and labetalol are effective in the management of SS by mitigation of autonomic dysregulation and sympathetic spells in patients with thalamic injury (15). SS is due to increased activity of the sympathetic nervous system (SNS) at the expense of the parasympathetic nervous system (PSNS) due to brain injury (16). The severity of traumatic brain injury (TBI) correlates with the level of sympathetic activation. The implication is that early use of β-blockers in TBI may attenuate the development of SS (17). Luostarinen et al., a retrospective study showed that TBI in Covid-19 patients did not affect disease severity (18). About 55% of hospitalized Covid-19 patients develop neurological signs (19). These signs may remain for about three months following SARS-CoV-2 infection, suggesting the development of latent brain injury (20).

**Figure 1:**
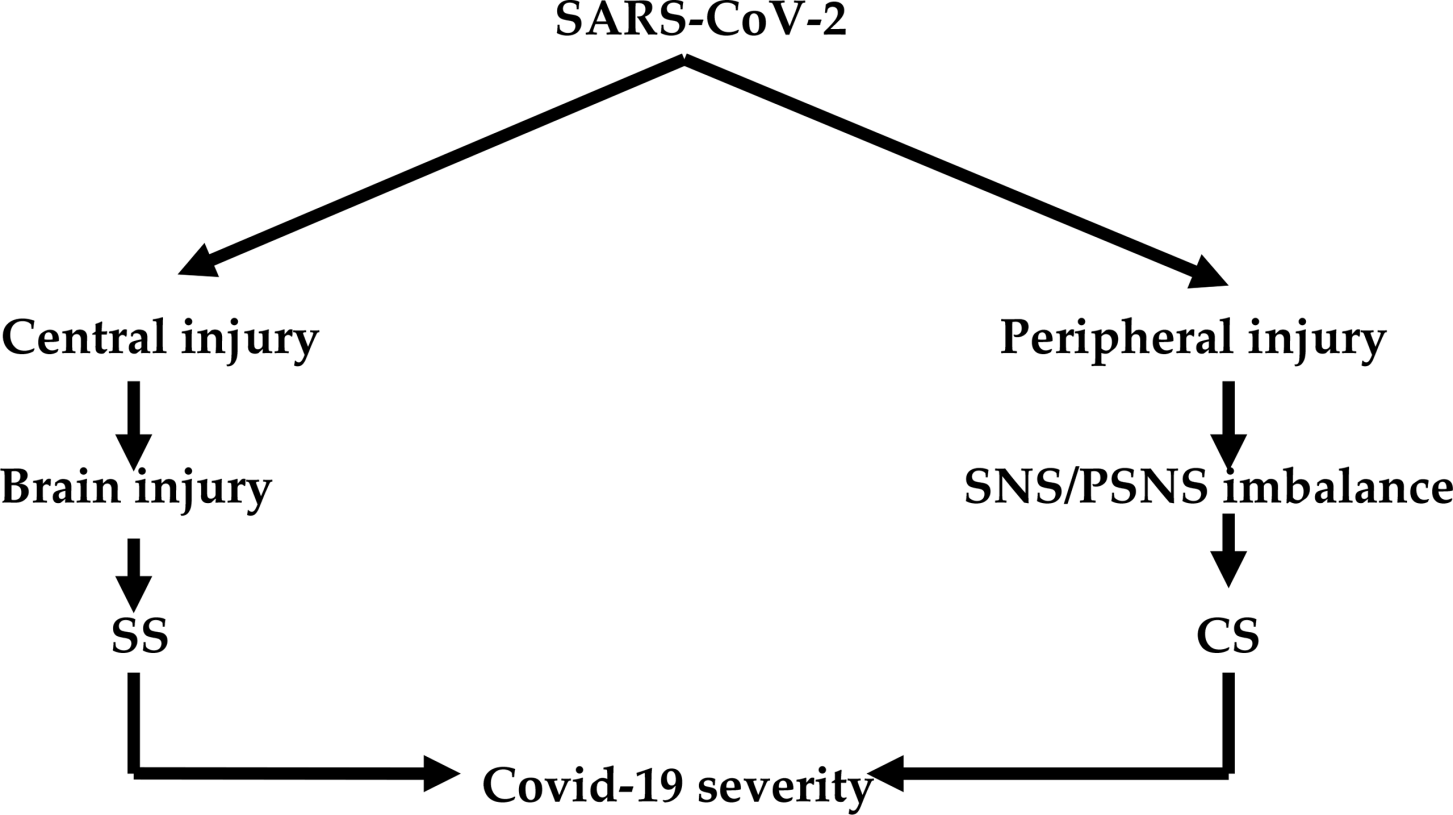
SARS-CoV-2 infection and development of sympathetic and cytokine storms: Central effect of SARS-CoV-2 leads to brain injury and development of sympathetic storm (SS). The peripheral effect of SARS-CoV-2 leads to induction imbalance between the sympathetic nervous system (SNS) and parasympathetic nervous system (PSNS) and development of cytokine storm (CS). Both SS and CS lead to Covid-19 severity.

Invasion of the central nervous system (CNS) by SARS-CoV-2 has remained speculative (21). However, brain injury in Covid-19 patients might be due to the direct effect of SARS-CoV-2. Covid-19 may lead to brain injury because it manifests with hypoxemia, autoimmune response, thrombosis, and CS (22). Notably, involvement of the peripheral nervous system (PNS) and autonomic nervous system (ANS) results in an imbalance between SNS and PSNS with the development of SS (23). The imbalance of the SNS/PSNS axis of ANS may affect the release of pro-inflammatory cytokines and immune-inflammatory response during Covid-19 (24). In this context, high circulating catecholamine levels may reflect sympathetic-mediated neutrophilia and T cell dysfunction in Covid-19 due to SS (25). Thus, the development SS in Covid-19 is through central and peripheral effects of SARS-CoV-2 that increase sympathetic outflow (**Figure 1**).

**Figure 2:**
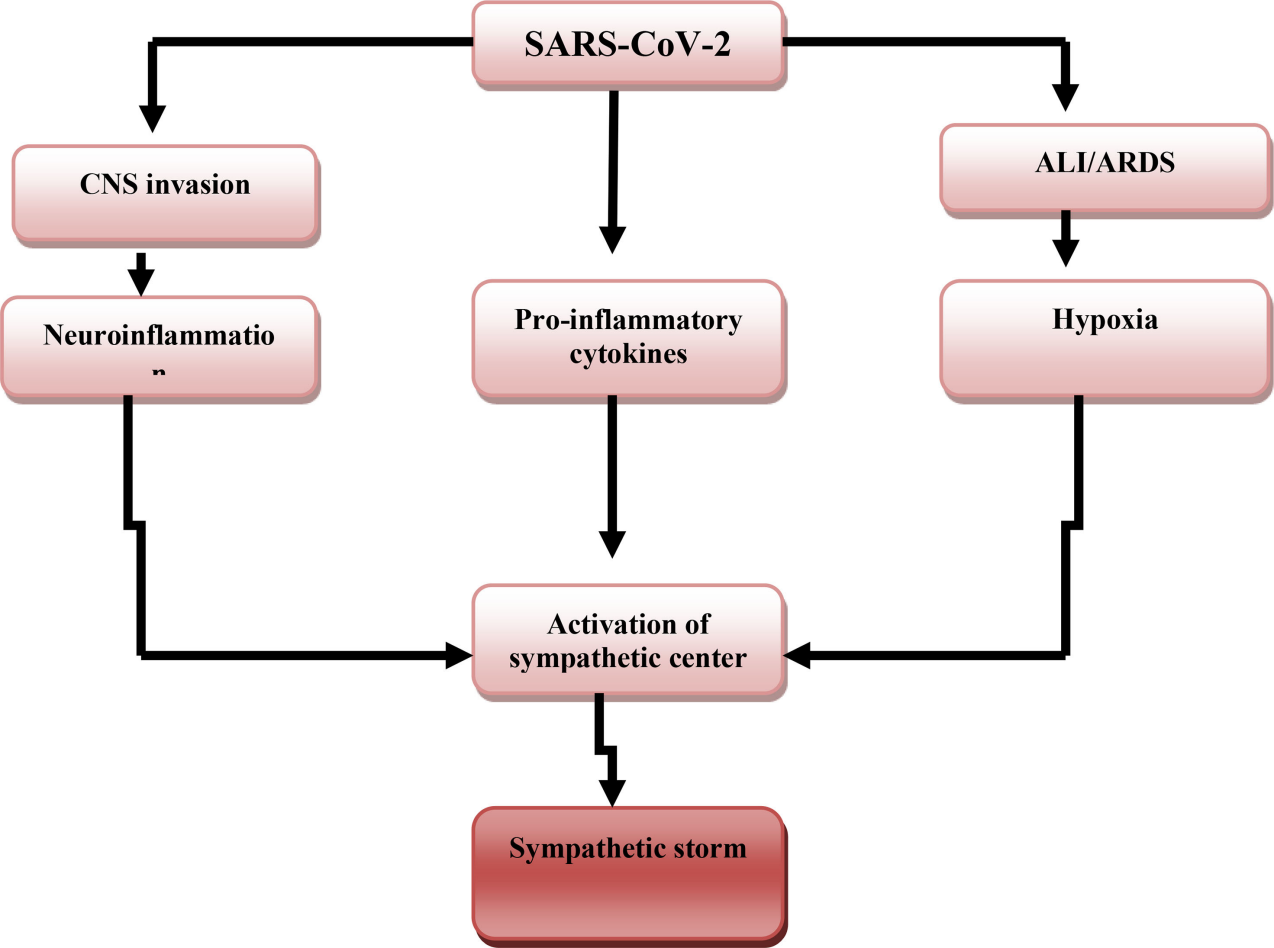
SARS-CoV-2-induced sympathetic storm. SARS-CoV-2 acute lung injury (ALI) and acute respiratory distress syndrome (ARDS)-induced hypoxia, SARS-CoV-2-induced neuroinflammation, and release of pro-inflammatory cytokines activate the central sympathetic center with the development of the sympathetic storm.

The potential mechanisms of SS in Covid-19 are through the three pathways including; ALI/ARDS-induced hypoxia, which activate the central sympathetic center through carotid bodies chemosensory input (26). SARS-CoV-2-induced neuroinflammation directly activates sympathetic centers like locus coeruleus (LC), rostral ventrolateral medulla (RVLM), and hypothalamic paraventricular nucleus (HPVN) (27). SARS-CoV-2-induced pro-inflammatory cytokines, which cross the blood-brain barrier and activate sympathetic center (**Figure 2**) (28).

**Figure 3:**
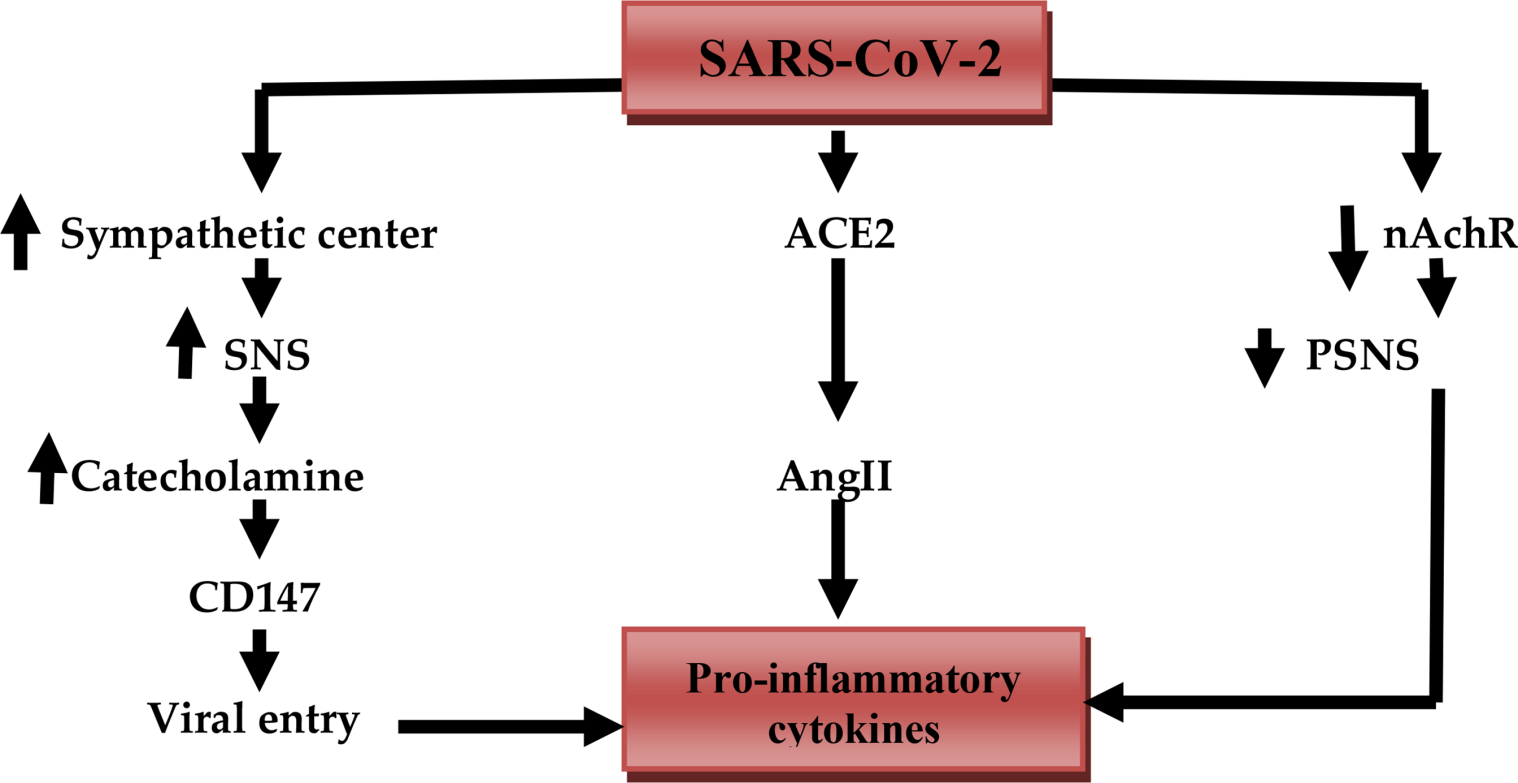
SARS-CoV-2 and release of pro-inflammatory cytokines: SARS-CoV-2 activates the sympathetic center, increases activity of the sympathetic nervous system (SNS), the release of catecholamine, which activates the expression of CD147 that increase viral entry. SARS-CoV-2 downregulates ACE2 which increases circulating angiotensin II (AngII). SARS-CoV-2 inhibits anti-inflammatory nicotinic acetylcholine receptor (nAChR) with reduced activity of the parasympathetic nervous system (PSNS). These changes together trigger the release of pro-inflammatory cytokines.

Moreover, comorbidities that induce a high sympathetic activity such as diabetes mellitus and hypertension may exacerbate cardiac arrhythmia, cardiac arrest, and acute myocardial infarction (29). Development of Covid-19 severity is linked with SS and vagal suppression that culminates into the CS (30). It is thus suggested that vagal stimulation might be valuable in Covid-19 patients through modulation of SS and release of pro-inflammatory cytokines (31). It has been shown that cholinergic agonists inhibit inflammation *via* suppression of inflammatory signals such as high mobility group protein 1 (HMGB1) (32). Furthermore, a molecular docking study observed that the nicotinic acetylcholine receptor (nAChR) may be a potential binding receptor for SARS-CoV-2 (32). Inhibition of nAChR by SARS-CoV-2 leads to inhibition of PSNS and exaggeration of SNS with subsequent progression of CS due to inhibition of vagal anti-inflammatory mediated by diminution of nAChR activity (33). Likewise, α-1 and β-receptor antagonists have valuable effects in Covid-19 *via* lessening of SS and development of CS (34). For that reason, β-blockers reduce sympathetic stimulation and inhibit the interaction between SARS-CoV-2 and receptor binding sites of angiotensin-converting enzyme 2 (ACE2) and CD147 (35) (**Figure 3**).

β-blockers reduce SS-induced cardiac arrhythmia, and destabilization of coronary plaques due to high circulating catecholamine, which cause positive inotropic and chronotropic effects through β1 receptor (36). As well, β-blockers reduce cardiac injury caused by sympathetic over-activation. Cardiomyocyte inflammation results from induction of local TNF-α and IL-6 expression (37).

Moreover, binding of SARS-CoV-2 to the ACE2 leads to deregulation of the renin-angiotensin system (RAS) with upregulation of vasoconstrictor angiotensin II (AngII). There is a co-current down-regulation of vasodilator Ang 1-7 leading to hypertension, sympathetic stimulation, and development of ALI and ARDS (38). β-blockers, therefore, reduce the activity of RAS by inhibiting the release of renin from renal juxtaglomerular cells, and protects the lungs and heart from exaggerated RAS and SS (39). An experimental study by Danukalo et al. illustrated that AngII increases firing and activity of LC with the propagation of sympathetic activation and hypertension in rats (40). Besides, β-blockers like propranolol modulate the activity and sensitivity of LC and prevent sympathetic stimulation in patients with migraines (41). Indeed, non-selective and lipophilic β-blockers like propranolol have a potent effect in the suppression of catecholamine from presynaptic adrenergic neurons through inhibition of excitatory presynaptic β2 autoreceptor (42). Taken together, β-blockers reduce the development of SS directly or indirectly through suppression of the central effect of AngII.

β-blockers prevent SS-induced ALI as high circulating catecholamines are linked with the development of ALI/ARDS (43). In addition, β-blockers prevent ALI through modulation of neutrophilia, lymphopenia, and the release of pro-inflammatory cytokines (44). In a retrospective study that involved 651 patients in ICU with sepsis, the patients on chronic β-blockers therapy had a lower risk of sepsis-induced ARDS. The patients required less mechanical ventilation due to upregulation of protective alveolar β2 adrenoceptors (45). Likewise in a randomized controlled clinical trial of 314 patients with acute respiratory failure in the ICU showed that patients on β-blockers therapy had a lower in-hospital mortality rate (46). Contrastingly, Mutlu et al., observed that β2-agonists improve alveolar fluid clearance in patients with pulmonary edema through up-regulation of alveolar epithelial sodium active transport (47). A study that involved 79 patients with ALI is associated with impairment of pulmonary alveolar clearance rate (48). These findings imply that selective β1-blockers are safer than non-selective ones in prevention of β2 adrenoceptors’ beneficial effect.

**Figure 4:**
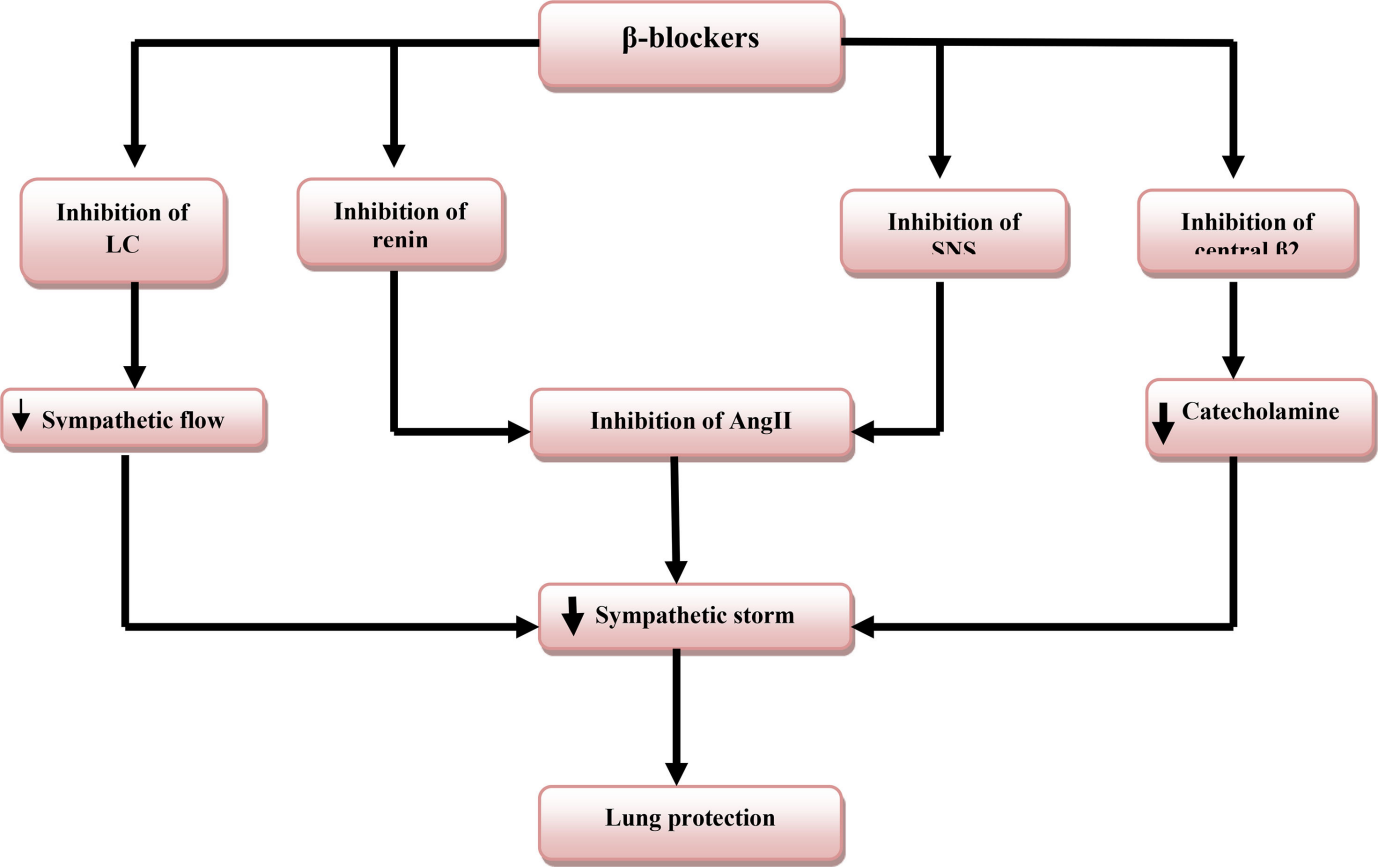
Role of β-blockers in lung protection: β-blockers inhibit sympathetic nervous system (SNS), renin release, locus coeruleus (LC) activity, and central presynaptic β2 receptors that decrease the release of catecholamine and angiotensin II (AngII) with subsequent inhibition of sympathetic storm and lung protection.

The findings support the favorable effects of β-blockers in the mitigation of SS-induced ALI/ARDS in severely affected Covid-19 patients (**Figure 4**).

## β-Blockers and Cytokine Storm in Covid-19

**Figure 5:**
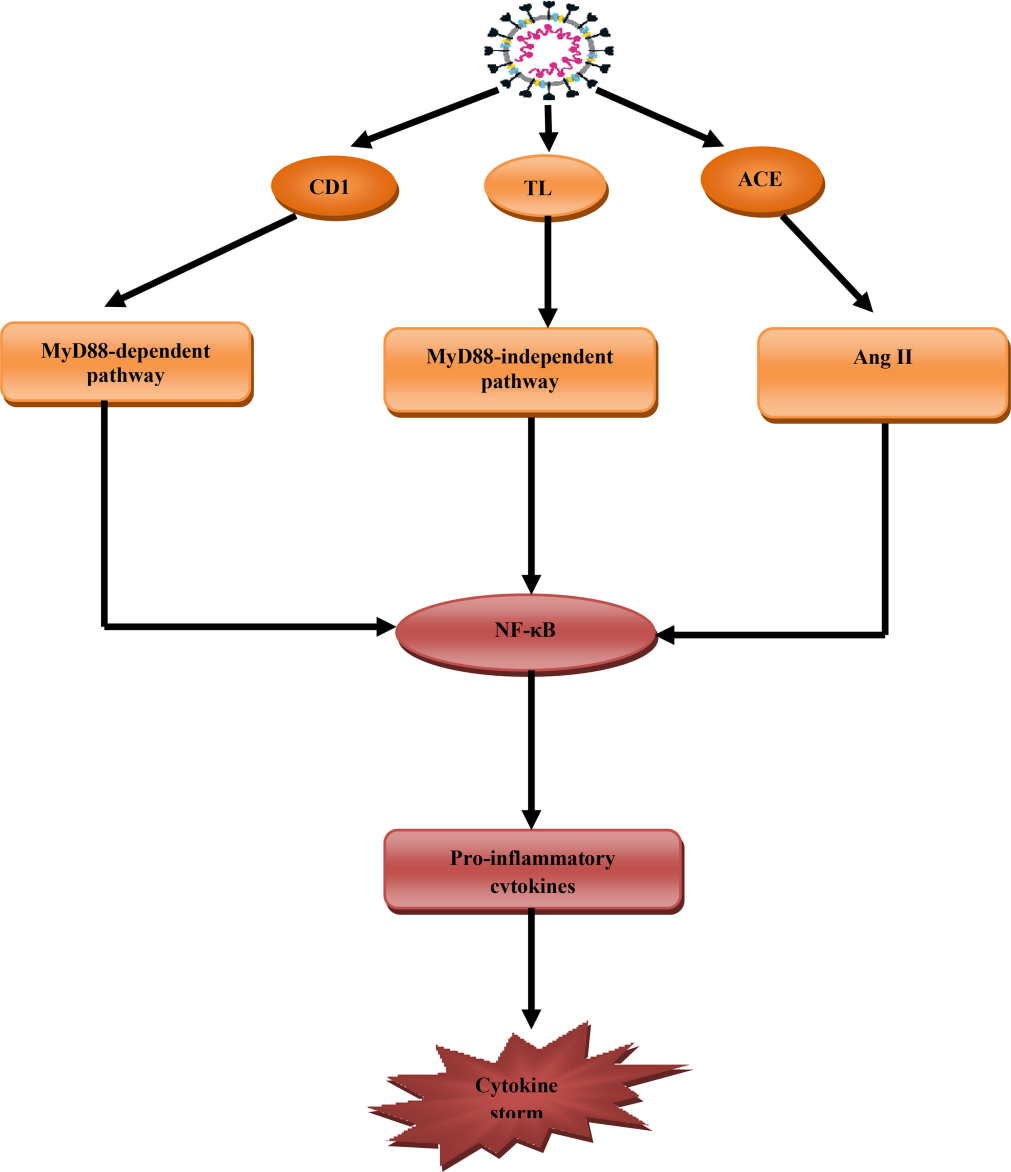
Role of SARS-CoV-2 in the development of cytokine storm (CS): SARS-CoV-2 through activation of CD147 activate myeloid differentiation 88 (MyD88), through toll-like receptor 4 (TLR4) and ACE2 activate angiotensin II (AngII) that together trigger NF-κB pathway, which stimulates the release of pro-inflammatory pathway and development of cytokine storm.

CS or cytokine releasing syndrome is a systemic inflammatory syndrome characterized by high circulating pro-inflammatory cytokines. CS also involves abnormal immunological hyperactivation involving pathogens, autoimmune reactions, and cancers (49). In Covid-19 pro-inflammatory cytokines including IL-6, TNF-α, IL-1β, and macrophage inflammatory protein (MIP) are elevated. Plasmablasts, CD4 and CD8, and other immune cells are also activated in CS (50). The interaction between SARS-CoV-2 and ACE2 on the affected cells induces cell damage. The interaction also causes the release of damage and inflammatory signals. The mentioned signals activate macrophages for the release of chemokines and pro-inflammatory cytokines that trigger T cells recruitment and activation (51). In addition, SARS-CoV-2 spike protein can activate CD147 and toll-like receptor 4 (TLR4) leading to stimulation of myeloid differentiation 88(MyD88) pathway. Myeloid differentiation provokes nuclear factor kappa B (NF-κB), which stimulates the release of pro-inflammatory cytokines and the development of CS (52) (**Figure 5**).

Interestingly, adrenergic receptors are linked with immunological disorders and the development of immune-mediated ALI since 90% of β-receptors are located in the lung alveoli with β2 predominant in 70% (53). β2 receptors are expressed by all immune cells especially macrophages, dendritic cells, and lymphocytes (54). Therefore, the β2-receptors signaling pathway play a crucial role in the production of pro-inflammatory cytokines, macrophage activation, and B-cells. The B-cells are involved in the production of antibodies which exacerbates the inflammation (55). Thus, β2-agonists may induce alveolar inflammation and pulmonary microvascular thrombosis *via* the accelerated release of IL-6 (56). Nossent et al. observed that β2-agonists increase the risk of venous thrombosis through activation of von Willebrand factor and factor VIII (57). In addition, activation of β2 receptors leads to the generation of reactive oxygen species (ROS) and induction of oxidative stress. Oxidative stress activates the release of IL-6, promotion of Th2 immune response, and inhibition of interferon-gamma (INF-γ) (58).

β-blockers have anti-inflammatory effects through reduction release of IL-6 and TNF-α, with inhibition of NF-κB and signal transduction and activator of transcription 3 (STAT3) (59). These pro-inflammatory cytokines and inflammatory signaling pathways are highly activated in Covid-19 in the progression of CS (60). Therefore, β-blockers may attenuate the development of CS in patients with severe Covid-19 (61). Additionally, β-blockers may reduce SARS-CoV-2-induced coagulopathy and pro-thrombotic complications through inhibition of platelet aggregations and factor VIII (62). β-blockers alleviate endothelial dysfunction and microvascular dysfunction linked with coagulopathy in Covid-19 through suppression of vascular endothelial growth factor (63).

CS is also developed due to activation of nod-like receptor pyrin 3 (NLRP3) inflammasome by SARS-CoV-2 viroporin (64). Gao et al. found that β-blocker nebivolol inhibits NLRP3 inflammasome in obesity-induced vascular remodeling in experimental animals (65). So, β-blockers could have potential benefits in mitigating the progression of SARS-CoV-2-mediated CS. SS with high catecholamine levels activates RAS with induction of AngII-mediated ALI and release of pro-inflammatory cytokines. Therefore β-blockers through inhibition of renin release and suppression of RAS may weaken the release of pro-inflammatory cytokines and the development of CS (66). Furthermore, macrophage activation syndrome (MAS) like disease is developed in severely affected Covid-19 leading to ALI, ARDS, and MOF (67). Xia et al. illustrated that high circulating catecholamine levels are associated with macrophages activation and release of pro-inflammatory cytokines (68). A prospective study involving 32 patients with immune-mediated dilated cardiomyopathy showed that β-blockers therapy reduces pro-inflammatory TNF-α. β-blockers increase anti-inflammatory IL-10 through inhibition of macrophage activation (69). Thus, β-blockers may reduce the development of MAS through inhibition of macrophage activation and release of pro-inflammatory cytokines (70). Nateasan preprinted study summarized the beneficial effects of β-blockers in Covid-19 in some points including that β-blockers improve oxygenation, reduce bronchial secretion, inhibit entry of SARS-CoV-2 through ACE2 and CD147, inhibit the release of pro-inflammatory cytokines, reduce the development of pulmonary edema and ARDS, inhibit the development of endothelial dysfunction and coagulopathy, block proliferation of SARS-CoV-2, and finally suppression of NLRP3 inflammasome and NF-κB signaling (62).

**Figure 6:**
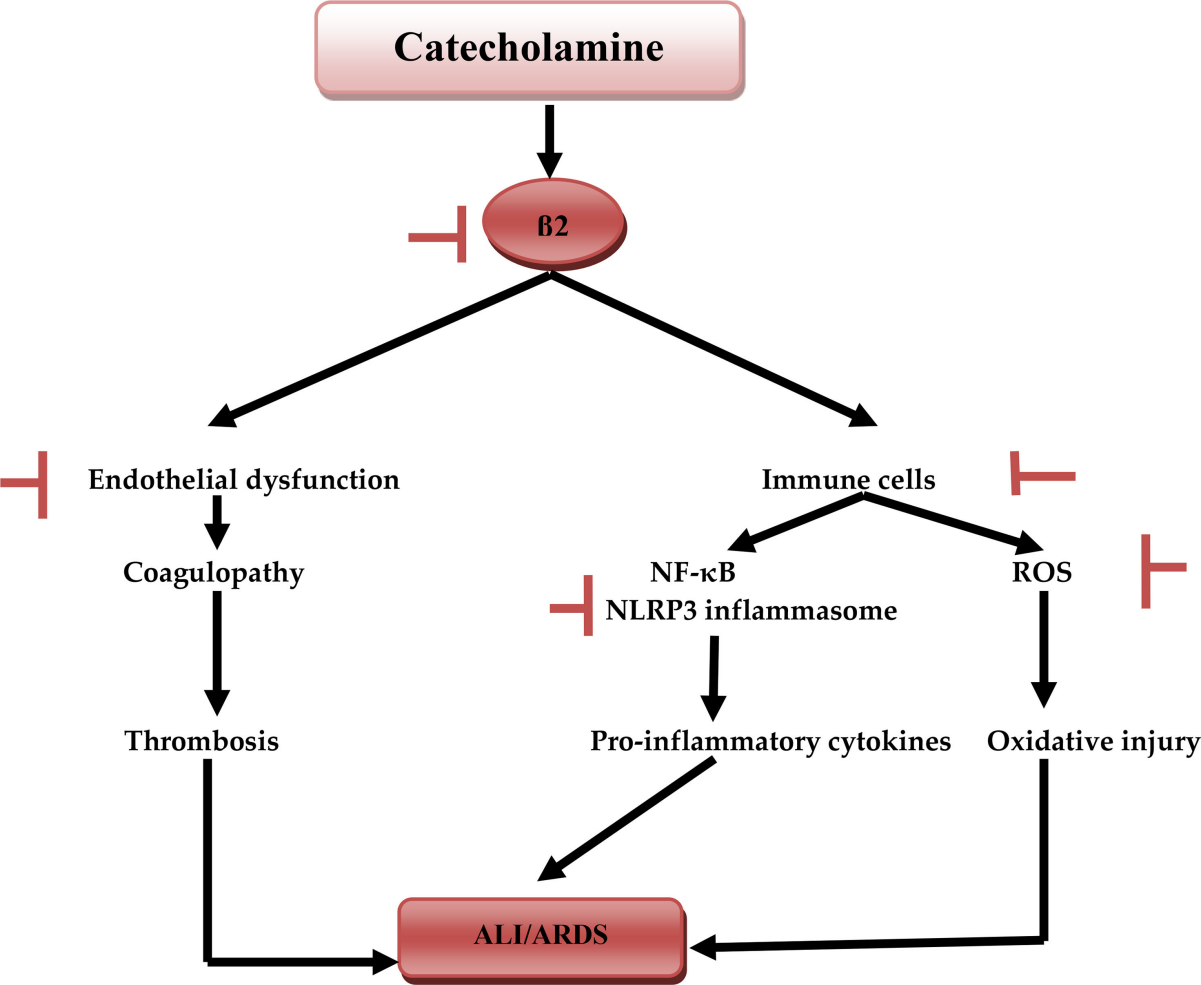
Catecholamine and acute lung injury: catecholamine during SARS-CoV-2-induced sympathetic storm, activates β1, which activates NF-κB and NLRP3 inflammasome of immune cells macrophages and neutrophils induces the release of pro-inflammatory cytokines. Activation of immune cells triggers the generation of reactive oxygen species (ROS), activation of β2 leads to the development of endothelial dysfunction, coagulopathy, and thrombosis. Together these changes cause acute lung injury (ALI) and acute respiratory syndrome (ARDS). Inhibitory effects of β-blockers.

Taken together, according to these findings, β-blockers might have a potential therapeutic modality in the prevention development of CS in Covid-19 (**Figure 6**).

## Neuroinflammation, Sympathetic and Cytokine Storms in Covid-19

Covid-19 causes systemic as well as different neurological disorders characterized by anosmia, headache, seizure, cerebrovascular complications, impaired consciousness, psychiatric disorders and dysautonomia (71). These neurological manifestations in Covid-19 are due to direct SARS-CoV-2 cytopathic effects or indirectly due to cerebral endothelial dysfunction and neuroinflammation caused by the cytokine storm (71). In addition, Covid-19 can aggravate neuroinflammation through activation of neurons, microglia, astrocytes and endothelial cells with subsequent increase of cytokines and chemokines in the CNS (72). Similarly, activations of dendritic cells, monocytes/macrophages, mast cells, endothelial and epithelial cells contribute to induction of neuroinflammation by releasing of pro-inflammatory cytokines and development of cytokine storm-induced neuroinflammation (73). Furthermore, Covid-19-induced psychological stress and sympathetic activation can exacerbate neuroinflammation *via* induction activation of mast cells and other immune cells to release pro-inflammatory cytokines (73). Therefore, there is a neuro-immunopathological mechanistic linkage between neuroinflammation with SS and CS in Covid-19 patients. It has been shown that neuroinflammation together with activated AngII and oxidative stress can provoke activation of sympathetic outflow with development of hypertension (74). Winklewski et al., observed that neuroinflammation plays a crucial role in the neuronal injury and activation of SNS. In turn, overactivated SNS induces pulmonary complications with increased risk of pneumonia, which causes a bystander autoimmune response against the CNS, thereby initiating a vicious cycle (75). Therefore, SARS-CoV-2-induced neuroinflammation could be the potential cause of sympathetic overactivity in severely affected Covid-19 patients (76). Sympathetic overactivity and development of SS may exert noteworthy detrimental effects on Covid-19 patients through action on the CNS and other vital organs (76). In turn, SARS-CoV-2 infection may increase sympathetic discharge through induction of CS, hypoxia and RAS imbalance (76). β-blockers mainly propranolol are effective in reduction of paroxysmal sympathetic overactivity following TBI (77). Furthermore, selective β-blockers can mitigate sympathetic overactivity and SS-induced neuroinflammation following ischemic stroke since up-regulated β2-adrenoceptor anti-inflammatory activity acts against post-stroke neuroinflammation (78). However, Evans et al., experimental study illustrated that β-blockers have pro-inflammatory effects so may increase neuroinflammation by inducing synaptic phagocytosis (79). The chronic use of β-blockers reduces systemic inflammation in patients with chronic liver disorders (80). Also, prolonged use of β-blockers in hypertensive Covid-19 patients does not affect the mortality but decreases risk for development of CS and SS (81). Taken together, these findings give a clue that β-blockers could have potential role in attenuation of neuroinflammation, SS and CS in Covid-19 patients.

## Cross talk Between Sympathetic and Cytokine Storms in Covid-19

It is proposed that cortical inhibitory GABAerigic neurons inhibit pre-sympathetic hypothalamic PVN neurons (82). These GABAerigic neurons have a high expression of ACE2 receptors. Therefore down-regulation of ACE2 receptors during SARS-CoV-2 infection may suppress these inhibitory inter-neurons with activation of hypothalamic PVN sympathetic neurons (83). Down-regulation of ACE2 during SARS-CoV-2 infection also augments AngII level, which has a potent stimulatory effect on the central hypothalamic PVN sympathetic neurons (84). Notably, central sympathetic stimulation due to SARS-CoV-2 infection increases circulating catecholamine. Catecholamines activate macrophages and neutrophils for the release of pro-inflammatory cytokines. Activated macrophages and neutrophils also release catecholamines, which act in a paracrine manner for augmentation of pro-inflammatory cytokines release (85). Rlddell speculated that catecholamine acts as a fuel for the activation and boosting of macrophages and neutrophils and the development of CS (86). Indeed, high catecholamine levels interact with pro-inflammatory cytokines in the progression of capillary leak syndrome and the development of MOF (87). These findings confirm the potential nexus between SS and CS in the development of MOF in patients with severe Covid-19 (88). An experimental study showed that interruption of catecholamine synthesis and release by metyrosine inhibits the development of CS in mice induced by T cell targeting antibodies (89).

Furthermore, high catecholamine levels during the development of SS in Covid-19 patients facilitate entry of SARS-CoV-2 *via* induction expression of CD147. Expressed CD147 causes damage to the lung alveolar basement membrane through activation of matrix metalloproteinase (MMPs) (90). In turn, alveolar membrane injury triggers the release of catecholamine from activated macrophages and neutrophils with the generation of the vicious cycle of injury (91). Thus, inhibition of CD147 may alleviate ALI through disruption of catecholamine-mediated acute inflammatory reactions (92). Hence, β-blockers may reduce pulmonary inflammation and alveolar dysfunction through inhibition of CD147 and MMPs in SARS-CoV-2 infection (93). Inhibition of CD147 leads to significant down-regulation of NF-κB signaling, which is the central pathway for the activation release of pro-inflammatory cytokines (94). Therefore, β-blockers through inhibition of CD147, NF-κB signaling and other inflammatory molecules (95) are potentially considered anti-inflammatory agents and may mitigate Covid-19 severity.

Moreover, CD147 play an important role in the progression of inflammatory and thrombotic pathways by triggering the interaction between platelets, immune and endothelial cells (96). Jin et al., experimental study demonstrated that thrombotic-induced acute ischemic stroke triggers expression and upregulation of CD147 in the brain micro-vessels and circulating platelets and leukocytes causing secondary microthrombosis (97). Therefore, inhibition of CD147 improves acute ischemic stroke by inhibiting thrombo-inflammation (97). CD147 could represent a novel therapeutic target against thrombo-inflammatory disorders. In addition, cyclophilin A stimulates platelets adhesion and thrombus formation through activation of CD147 (98). Thus, inhibition of the cyclophilin A-CD147 interaction attenuates acute pulmonary thrombosis in experimental rats (99). In SARS-CoV-2 infection both cyclophilin A and CD147 are activated with substantial development of pulmonary micro-thrombosis (100, 101). In addition, activation of cyclophilin A and CD147 is associated with cardiac inflammation and fibrosis in patients with heart failure (102). β-blockers improve cardiac function in patients with heart failure *via* inhibiting expression of cyclophilin A and CD147 (102). Furthermore, CD147 induces expression of vascular endothelial growth factor (VEGF) in different inflammatory disorders (103). Yin et al., found that high VEGF serum levels are linked with neuroinflammation through recruitment of inflammatory cells and activation of brain AngII in Covid-19 patients (104), while Barbieri et al., showed that high endothelial nitric oxide synthase levels associated with VEGF activity in stressed mice (105). It has been reported that β-blockers can reduce expression of VEGF in patients with lung cancer (71). Therefore, β-blockers are able to inhibit development of pulmonary thrombo-embolism and SARS-CoV-2 infection-induced heart failure by inhibiting cyclophilin A-CD147 expression in the platelets and cardiomyocytes respectively with attenuation of neuroinflammation *via* inhibition of VEGF.

It has also been proposed that α1-blockers like prazosin are effective in the mitigation of CS in Covid-19 through inhibition release of IL-6 (106). Therefore, dual β and α1-blocker like labetalol might be more effective in suppressing the development of CS through complete blocking of catecholamine effects on the immune cells during SS in Covid-19 (107).

**Figure 7:**
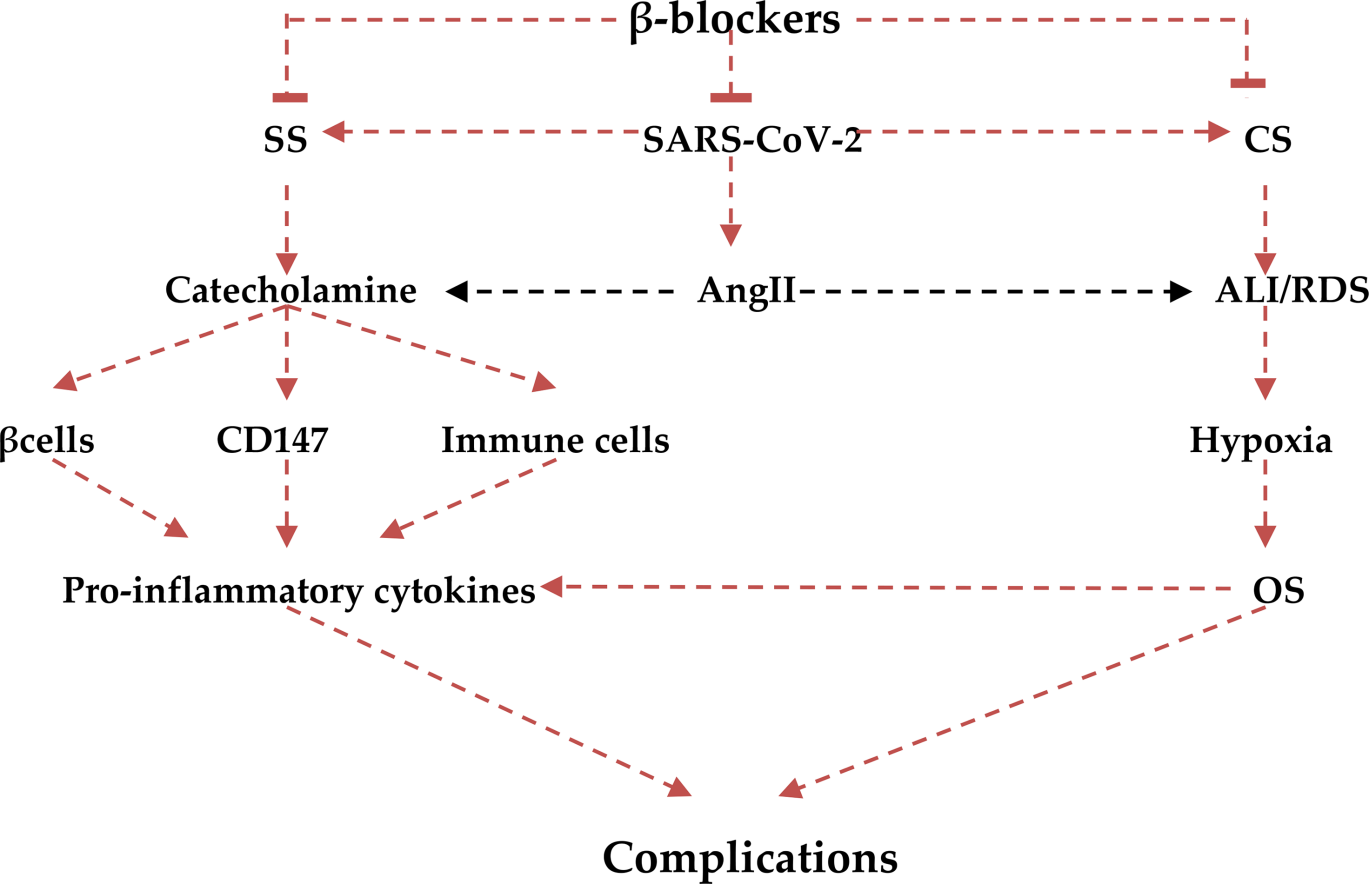
Role of β-blockers in the irruptions of the crosstalk between cytokine (CS) and sympathetic storms(SS): β-blockers block reduces the release of catecholamine and decreases its stimulatory effect on β2, CD147, and immune cells with reduction release of pro-inflammatory cytokines. The anti-inflammatory effects of β-blockers also attenuate CS-induced acute lung injury (ALI) and acute respiratory syndrome (ARDS), development of oxidative stress (OS), and final systemic complications.

Interestingly, β-blockers mainly carvedilol has anti-oxidant effects that are induced by high catecholamine level in patients with heart failure (108). Therefore, β-blockers block the development of oxidative stress during the development of SS and CS in Covid-19 that is associated with various complications like endothelial dysfunction and coagulopathy (109). It has been shown that toxic gas-induced pulmonary alveolar membrane injury triggers cascades for the development of oxidative stress. Oxidative stress then provokes neutrophils and macrophages to release pro-inflammatory cytokines and the development of ALI (110). Similarly, oxidative stress injury in SARS-CoV-2 infection escalates the release of pro-inflammatory cytokines in an oxidative-dependent manner in the development of CS (111). Notably, myeoloperoxidase (MPO) is regarded as a linking marker between oxidative stress and inflammation. High MPO activity is linked with the development of cardiovascular complications (112). MPO is regarded as a natural immune response, which releases hypochloruous acid (HOCI). HOCL competes for oxygen binding at heme molecule of hemoglobin causing heme destruction and release of free iron that cause acute tissue injury through the generation of ROS in Covid-19 (113). This confirms that MPO-induced oxidative stress is regarded as the chief central pathway linking the development of CS and SS in Covid19. β-blockers mostly metoprolol block the activity of MPO and mitigate the development of oxidative stress and further development of sympoatho-cytokine storm (108). Therefore, there is considerable crosstalk between SS and CS in Covid-19 (**Figure 7**).

The present study revealed β-blockers are effective mitigators of both SS and CS through interruption of catecholamine-β receptors interaction and inhibition release of pro-inflammatory cytokine and development of CS in Covid-19. Despite these beneficial effects of β-blockers in Covid-19 patients, β-blockers have some adverse effects including bradycardia, bronchospasm, peripheral vasoconstrictions, insomnia and depression (114). These adverse effects of β-blockers mainly bronchospasm and peripheral vasoconstrictions may adversely affect the pulmonary function in aged critically ill Covid-19 patients. However, selective β-blockers like celiprolol, nebivolol and carvedilol have less adverse effects due to their selectivity against β1-adrenoceptors (114). Moreover, celiprolol, which is β1-adrenoceptors antagonist and β2-adrenoceptors agonist, is safe in asthmatic patients as it induces bronodilation (115). However, β2-adrenoceptors which expressed on airways and immune cells contribute to exaggerated immune response in the early phase of SARS-CoV-2 infection (61). β2-adrenoceptors on the alveolar macrophages promote secretion of IL-6 and induction of immuno-thrombosis with development of pulmonary thromboembolism (61). Thus, non-selective β-blockers counteract pulmonary thromboembolism by inhibiting prothrombotic response, secretion of VEGF and vascular tone (116). Futhermore, selective inhibitors of β2-adrenoceptors (ICI) blocks the fosforilation of NfKB principal orchestrator of inflammatory response (117).

Therefore, use of non-selective β-blockers in Covid-19 patients in the early phase could be beneficial to attenuate SARS-CoV-2 infection-induced hyperinflammation through inhibition pro-inflammatory cytokines release (61). Likewise, non-selective β-blockers block exaggerated immune response and pulmonary thrombosis by suppressing Th17 activation and procoagulant status respectively. Therefore, despite the adverse effects of β-blockers, non-selective β-blockers appear to be more effective than selective β-blockers in the clinical setting of Covid-19.

We lacked access to clinical data to enforce the concept, however, this study proposes a mechanism of cross-talk between CS and SS in Covid-19 regarding the potential role of β-blockers to guide further studies.

## Conclusion

The anti-inflammatory effect of β-blockers through the inhibition release of pro-inflammatory cytokines contributes to the mitigation of CS progression. In addition, β-blockers attenuate the development of SS due to SARS-CoV-2 infection-induced catecholamine release and sympatho-excitation. CS and SS interact at various levels to cause lethal complications in patients with severe COVID-19 like ALI, ARDS, and MOF. However, β-blockers interrupt this interaction through inhibition of several mediators of CS and SS. β-blockers also prevent the development of the neural-cytokine loop in SARS-CoV-2 infection. Evidence from this study triggers an idea for prospective studies to confirm the potential role of β-blockers in the management of Covid-19 in clinical trials.

## Author Contributions

HA-k, KK, GM-h, and GB conceptualized the study. HA-k, AA-G, KK, GZ, SW, and GB developed the study design. HA-k, AA-G, GM-h, KK, GZ, AA, MA, SW, and GB collected the data and analyzed it for scientific content. HA-k, KK, GM-h, SW, GB drafted the initial manuscript and revised it for intellectual content while HA-k, AA-G, GM-h, KK, GZ, AA, MA, SW, and GB approved the final version for publication and remain accountable on all aspects of the work therein. All authors contributed to the article and approved the submitted version.

## Funding

This work was funded by the Deanship of Scientific Research at Jouf University under grant Number DSR-2021-01-0374.

## Conflict of Interest

The authors declare that the research was conducted in the absence of any commercial or financial relationships that could be construed as a potential conflict of interest.

## Publisher’s Note

All claims expressed in this article are solely those of the authors and do not necessarily represent those of their affiliated organizations, or those of the publisher, the editors and the reviewers. Any product that may be evaluated in this article, or claim that may be made by its manufacturer, is not guaranteed or endorsed by the publisher.
